# Activities and genetic interactions of fission yeast Aps1, a Nudix-type inositol pyrophosphatase and inorganic polyphosphatase

**DOI:** 10.1128/mbio.01084-24

**Published:** 2024-06-28

**Authors:** Shreya Ghosh, Ana M. Sanchez, Beate Schwer, Isabel Prucker, Nikolaus Jork, Henning J. Jessen, Stewart Shuman

**Affiliations:** 1 Molecular Biology Program, Sloan Kettering Institute, New York, New York, USA; 2 Gerstner Sloan Kettering Graduate School of Biomedical Sciences, New York, New York, USA; 3 Department of Microbiology and Immunology, Weill Cornell Medical College, New York, New York, USA; 4 Institute of Organic Chemistry, University of Freiburg, Freiburg, Germany; 5 CIBSS - Centre for Integrative Biological Signalling Studies, University of Freiburg, Freiburg, Germany; Harvard Medical School, Boston, Massachusetts, USA

**Keywords:** inositol pyrophosphatase, inorganic polyphosphatase, Nudix hydrolase, phosphate homeostasis, *Schizosaccharomyces pombe*

## Abstract

Inositol pyrophosphate 1,5-IP_8_ regulates expression of a fission yeast phosphate homeostasis regulon, comprising phosphate acquisition genes *pho1*, *pho84*, and *tgp1*, via its action as an agonist of precocious termination of transcription of the upstream lncRNAs that repress *PHO* mRNA synthesis. 1,5-IP_8_ levels are dictated by a balance between the Asp1 N-terminal kinase domain that converts 5-IP_7_ to 1,5-IP_8_ and three inositol pyrophosphatases—the Asp1 C-terminal domain (a histidine acid phosphatase), Siw14 (a cysteinyl-phosphatase), and Aps1 (a Nudix enzyme). In this study, we report the biochemical and genetic characterization of Aps1 and an analysis of the effects of Asp1, Siw14, and Aps1 mutations on cellular inositol pyrophosphate levels. We find that Aps1’s substrate repertoire embraces inorganic polyphosphates, 5-IP_7_, 1-IP_7_, and 1,5-IP_8_. Aps1 displays a ~twofold preference for hydrolysis of 1-IP_7_ versus 5-IP_7_ and *aps1*∆ cells have twofold higher levels of 1-IP_7_ vis-à-vis wild-type cells. While neither Aps1 nor Siw14 is essential for growth, an *aps1*∆ *siw14*∆ double mutation is lethal on YES medium. This lethality is a manifestation of IP_8_ toxicosis, whereby excessive 1,5-IP_8_ drives derepression of *tgp1,* leading to Tgp1-mediated uptake of glycerophosphocholine. We were able to recover an *aps1*∆ *siw14*∆ mutant on ePMGT medium lacking glycerophosphocholine and to suppress the severe growth defect of *aps1*∆ *siw14*∆ on YES by deleting *tgp1*. However, the severe growth defect of an *aps1*∆ *asp1-H397A* strain could not be alleviated by deleting *tgp1*, suggesting that 1,5-IP_8_ levels in this double-pyrophosphatase mutant exceed a threshold beyond which overzealous termination affects other genes, which results in cytotoxicity.

**IMPORTANCE:**

Repression of the fission yeast *PHO* genes *tgp1*, *pho1*, and *pho84* by lncRNA-mediated interference is sensitive to changes in the metabolism of 1,5-IP_8_, a signaling molecule that acts as an agonist of precocious lncRNA termination. 1,5-IP_8_ is formed by phosphorylation of 5-IP_7_ and catabolized by inositol pyrophosphatases from three distinct enzyme families: Asp1 (a histidine acid phosphatase), Siw14 (a cysteinyl phosphatase), and Aps1 (a Nudix hydrolase). This study entails a biochemical characterization of Aps1 and an analysis of how Asp1, Siw14, and Aps1 mutations impact growth and inositol pyrophosphate pools *in vivo*. Aps1 catalyzes hydrolysis of inorganic polyphosphates, 5-IP_7_, 1-IP_7_, and 1,5-IP_8_ *in vitro*, with a ~twofold preference for 1-IP_7_ over 5-IP_7_. *aps1*∆ cells have twofold higher levels of 1-IP_7_ than wild-type cells. An *aps1*∆ *siw14*∆ double mutation is lethal because excessive 1,5-IP_8_ triggers derepression of *tgp1*, leading to toxic uptake of glycerophosphocholine.

## INTRODUCTION

**Fig 1:**
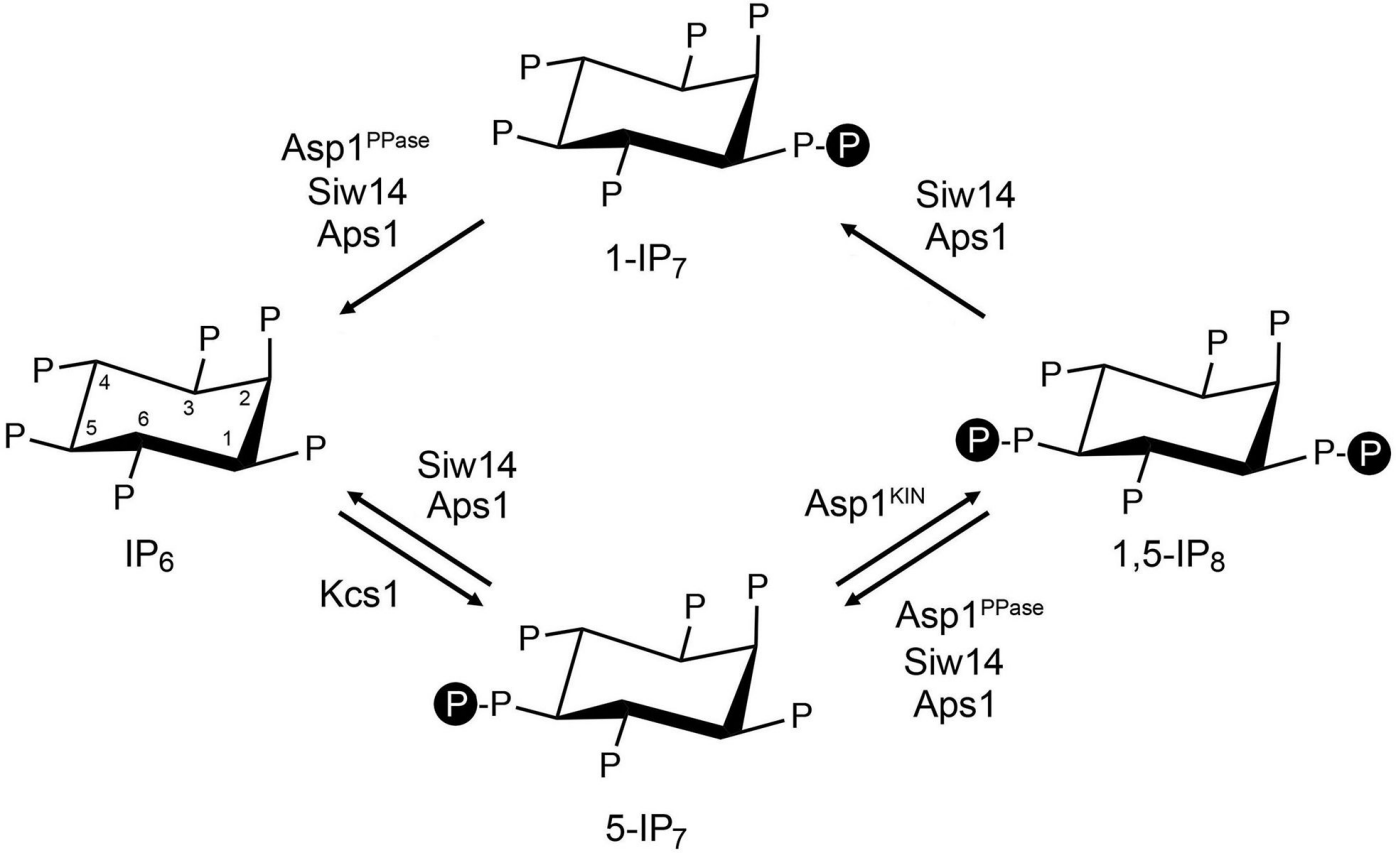
Inositol pyrophosphate metabolism in fission yeast. The chemical structures of IP_6_, 5-IP_7_, 1,5-IP_8_, and 1-IP_7_ are shown, with “P” denoting phosphate. The positions of the myo-inositol ring are indicated for IP_6_. The fission yeast enzymes that add (Kcs1 and Asp1-kinase) or remove (Siw14, Asp1-pyrophosphate, and Aps1) β-phosphate groups are indicated.

Inositol pyrophosphates IP_7_ and IP_8_ are eukaryal signaling molecules that influence phosphate and polyphosphate homeostasis (1). The isomers 5-IP_7_ and 1-IP_7_ differ as to whether the pyrophosphate moiety is at the 1 or 5 position of the inositol ring (Fig. 1). 1,5-IP_8_ is pyrophosphorylated at both positions. 1,5-IP_8_ is synthesized by the sequential action of kinases Kcs1/IP6K, which converts IP_6_ to 5-IP_7_, and Asp1/Vip1/VIH/PPIP5K, which converts 5-IP_7_ to 1,5-IP_8_ (1) (Fig. 1). Asp1, Vip1, VIH, and PPIP5K—in fission yeast, budding yeast, plants, and humans, respectively—are bifunctional enzymes composed of an N-terminal kinase domain that synthesizes 1,5-IP_8_ and a C-terminal pyrophosphatase domain, of the histidine acid phosphatase enzyme family, that hydrolyzes 1,5-IP_8_ back to 5-IP_7_ (2–8).

In addition to the pyrophosphatase domain of Asp1/Vip1/VIH/PPIP5K, two other classes of pyrophosphatases—Siw14 and DIPP/Ddp1/Aps1 (from human, budding yeast, and fission yeast, respectively)—are implicated in the catabolism of inositol pyrophosphates. Siw14 belongs to the cysteinyl-phosphatase family of metal-independent phosphohydrolases, defined by a conserved active site phosphate-binding loop HCxxxxxR, that catalyze phosphoryl transfer to water via a covalent enzyme-(cysteinyl-S)-phosphate intermediate. Budding yeast Siw14 specifically removes the 5-β-phosphate from 5-IP_7_ and 1,5-IP_8_ but does not hydrolyze the 1-β-phosphate of 1-IP_7_ (9, 10). The plant *Arabidopsis thaliana* encodes five paralogous Siw14 homologs (named PFA-DSPs 1–5) that prefer to hydrolyze the 5-β-phosphate of inositol pyrophosphates (11). By contrast, fission yeast Siw14 is adept at converting 5-IP_7_, 1-IP_7_, and 1,5-IP_8_ to IP_6_, without significant positional bias as to the β-phosphate hydrolyzed (12) (Fig. 1).

DIPP/Ddp1/Aps1 enzymes belong to the Nudix-family of metal-dependent pyrophosphohydrolases defined by a ~23-aa Nudix box motif in which three glutamates comprise a binding site for catalytic magnesium ions (13–16). DIPP/Ddp1/Aps1 were initially characterized as hydrolases acting on diadenosine polyphosphates Ap_6_A and Ap_5_A and on inositol pyrophosphates (13, 14, 17). *Saccharomyces cerevisiae* Ddp1 was subsequently shown to have vigorous endopolyphosphatase activity on linear inorganic polyphosphate (poly-P) substrates (18). Whereas Ddp1 hydrolyzes the β-phosphate from 1-IP_7_ to form IP_6_, 5-IP_7_ is a poor substrate for Ddp1 (15, 18). When presented simultaneously with poly-P and 1-IP_7_, Ddp1 preferentially hydrolyzed poly-P (18). Human DIPP enzymes also have poly-P endopolyphosphatase activity (18). Although initial kinetic analyses of human DIPP1 indicated that *k*_cat_ is an order of magnitude greater for 1-IP_7_ than for 5-IP_7_ or 1,5-IP_8_ (15), it was reported subsequently that human DIPP1 hydrolyzed 1-IP_7_ twice as fast as 5-IP_7,_ but (in contrast to the previous findings) 1,5-IP_8_ was hydrolyzed even faster than 1-IP_7_ (16). Mechanistically informative crystal structures have been reported for DIPP1 in on-pathway complexes with three catalytic magnesium ions and either 5-IP_7_ or IP_6_ (16, 19) and for Ddp1 in complexes with 5-IP_7_ and poly-P_15_ (20).

The present study is focused on the *Schizosaccharomyces pombe* Nudix enzyme Aps1, a 210-aa protein homologous to DIPP1 and Ddp1. Although Aps1 is inessential for vegetative growth, it plays a role in cellular phosphate homeostasis via its impact on IP_8_ dynamics. The fission yeast phosphate acquisition (*PHO*) genes *pho1* (cell surface acid phosphatase), *pho84* (phosphate transporter), and *tgp1* (glycerophosphodiester transporter) are repressed under phosphate-replete conditions by upstream lncRNA-mediated transcriptional interference (21) and derepressed during phosphate starvation when synthesis of the interfering lncRNAs is turned off (22, 23). Transcription of the upstream *PHO* lncRNAs interferes with the downstream *PHO* mRNA genes by displacing the activating transcription factor Pho7 from its binding site(s) in the mRNA promoters that overlap the lncRNA transcription units. *PHO* lncRNA 3'-processing and termination is a key control point in *PHO* mRNA repression; i.e., transcriptional interference can be tuned by increasing or decreasing the frequency with which Pol2 terminates lncRNA transcription prior to encounter with the mRNA promoter (21). Genetic maneuvers that enhance precocious termination of lncRNA transcription result in derepression of *PHO* mRNA expression in phosphate-replete cells, and those that reduce the probability of lncRNA termination prior to the mRNA promoter result in hyper-repression of the flanking *PHO* mRNAs relative to their basal levels.

The initial insights that inositol pyrophosphates are involved in fission yeast phosphate homeostasis were as follows: (i) deletion or active site mutations of the Asp1 kinase that synthesizes IP_8_ hyper-repress *pho1* under phosphate-replete conditions; (ii) inactivating mutations of the Asp1 pyrophosphatase domain or deletion of the Aps1 Nudix pyrophosphatase derepresses *PHO* genes under phosphate-replete conditions; and (iii) derepression of Pho1 by *aps1*∆ depends on synthesis of IP_8_ by the Asp1 kinase (24–26). Simultaneous inactivation of the Asp1 and Aps1 pyrophosphatases is synthetically lethal (26), as is simultaneous deletion of *aps1*^+^ and *siw14*^+^ (12); i.e., we were unable to recover viable *asp1-H397A aps1*∆ or *siw14*∆ *aps1*∆ haploid progeny on YES medium after crossing the respective single mutants. These results signify that too much IP_8_ is toxic to fission yeast. Multiple lines of genetic, biochemical, and transcriptomic evidence cohere to show that (i) IP_8_ acts as an agonist of precocious *PHO* lncRNA transcription termination dependent on the 3' cleavage and polyadenylation factor (CPF) complex (26–30) and (ii) IP_8_ toxicosis caused by Asp1 pyrophosphatase-inactivating mutations (so-called *asp1-STF* alleles isolated in a genetic suppressor screen for relief of lncRNA-mediated transcriptional interference with *pho1* expression) results from overexpression of the *tgp1* gene and deleterious import of glycerophosphocholine (GPC) present in the growth medium (31). Moreover, pyrophosphatase-defective alleles *aps1*∆ and *asp1-H397A* display severe synthetic growth defects in combination with *seb1-G476S*, a mutation of the essential transcription termination factor Seb1, which results in derepression of *pho1* and *tgp1* mRNAs by enhancing precocious termination of the upstream interfering lncRNAs (32).

In this study, we present a biochemical and genetic characterization of *S. pombe* Aps1. We report that (i) recombinant Aps1 catalyzes magnesium-dependent hydrolysis of a linear inorganic polyphosphate chain (poly-P) to generate tripolyphosphate as an end-product and (ii) Aps1 cleaves the β-phosphate moieties from 5-IP_7_, 1-IP_7_, and 1,5-IP_8_ to yield IP_6_ as an end-product (Fig. 1). While an E89A–E93A mutation in the metal-binding site of the Nudix motif abolished Aps1 polyphosphatase activity *in vitro*, the catalytically defective *aps1-(E89A–E93A*) mutant retained partial biological activity in complementing the lethality of *siw14*∆ *aps1*∆ on YES medium. The growth defects of *siw14*∆ *aps1*∆, *aps1∆ seb1-G476S*, and *asp1-H397A seb1-G476S* on YES medium are alleviated by deletion of the GPC transporter Tgp1. These results fortify emerging evidence that Tgp1 overexpression is a major contributor to IP_8_ toxicosis in fission yeast.

We also analyzed the effects of Aps1, Siw14, and Asp1 mutations on cellular levels of 5-IP_7_, 1-IP_7_, and 1,5-IP_8_ via capillary electrophoresis electrospray ionization mass spectrometry (CE-ESI-MS) (33, 34). Our findings complement and extend those of previous studies of fission yeast inositol pyrophosphate dynamics that relied on metabolic labeling of cells with ^3^H-inositol (3, 4).

## RESULTS

### Recombinant *S. pombe* Aps1 is a metal-dependent inorganic polyphosphatase

**Fig 2:**
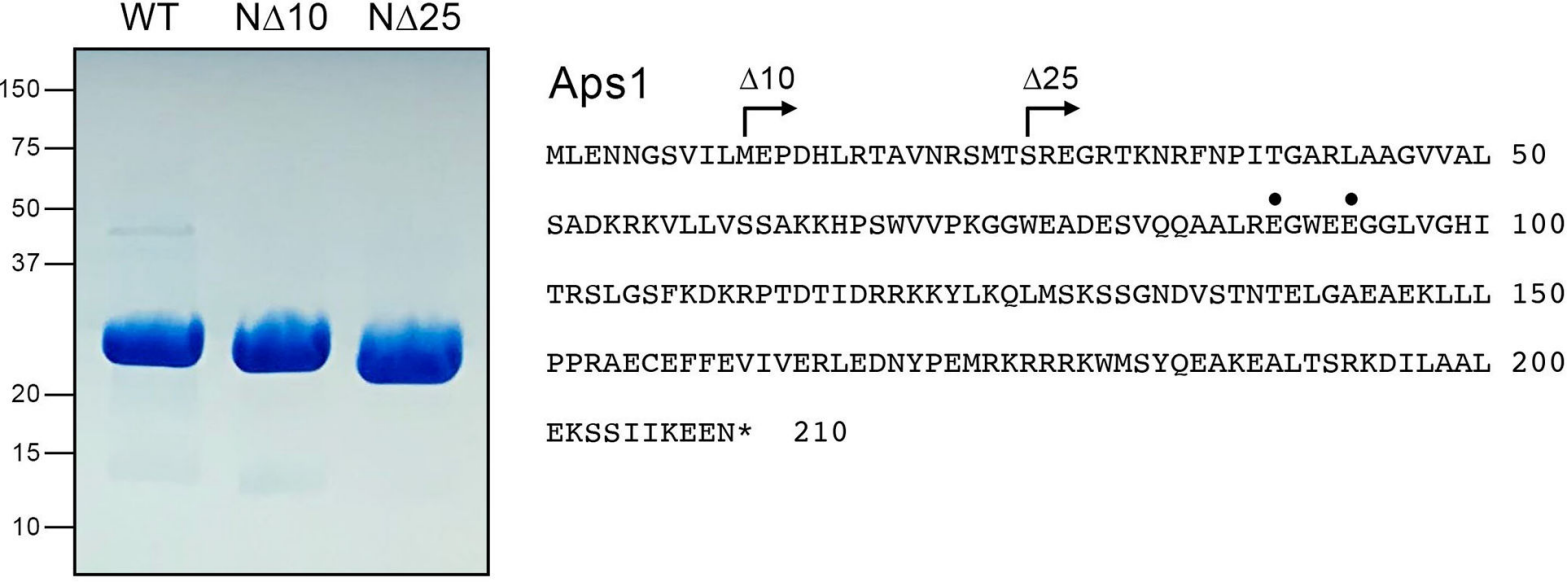
Recombinant full-length Aps1 and N∆ truncations. (Left panel) Aliquots (10 µg) of the Superdex 200 preparations of full-length Aps1 and the N∆10 and N∆25 truncated proteins were analyzed by SDS-PAGE. The Coomassie blue-stained gel is shown. The positions and sizes (kDa) of marker polypeptides are indicated on the left. (Right panel) Amino acid sequence of fission yeast Aps1. The margins of the truncations are indicated by arrows. The metal-binding glutamates E89 and E93 that were mutated to alanine are denoted by dots.

We produced recombinant full-length Aps1 (amino acid sequence shown in Fig. 2) in *Escherichia coli* as a His_10_Smt3 fusion and isolated His_10_Smt3-Aps1 from a soluble bacterial extract by Ni-affinity chromatography. The tag was removed by treatment with the Smt3 protease Ulp1, and the Aps1 protein was recovered free of His_10_Smt3 after a second round of Ni-affinity chromatography. In parallel, we produced and purified N-terminally truncated versions: N∆10 [Aps1-(11-210)] and N∆25 [Aps1-(26-210)] (Fig. 2). These truncations were made in light of the AlphaFold tool prediction that the N-terminal 25-aa segment of Aps1 does not adopt a definite secondary structure (https://alphafold.ebi.ac.uk/entry/Q09790). The tag-free Aps1 proteins were subjected to a final gel filtration step, during which they eluted as single peaks consistent with monomeric native size. SDS-PAGE revealed comparable purity of the Aps1 full-length and N∆ proteins (Fig. 2).

**Fig 3:**
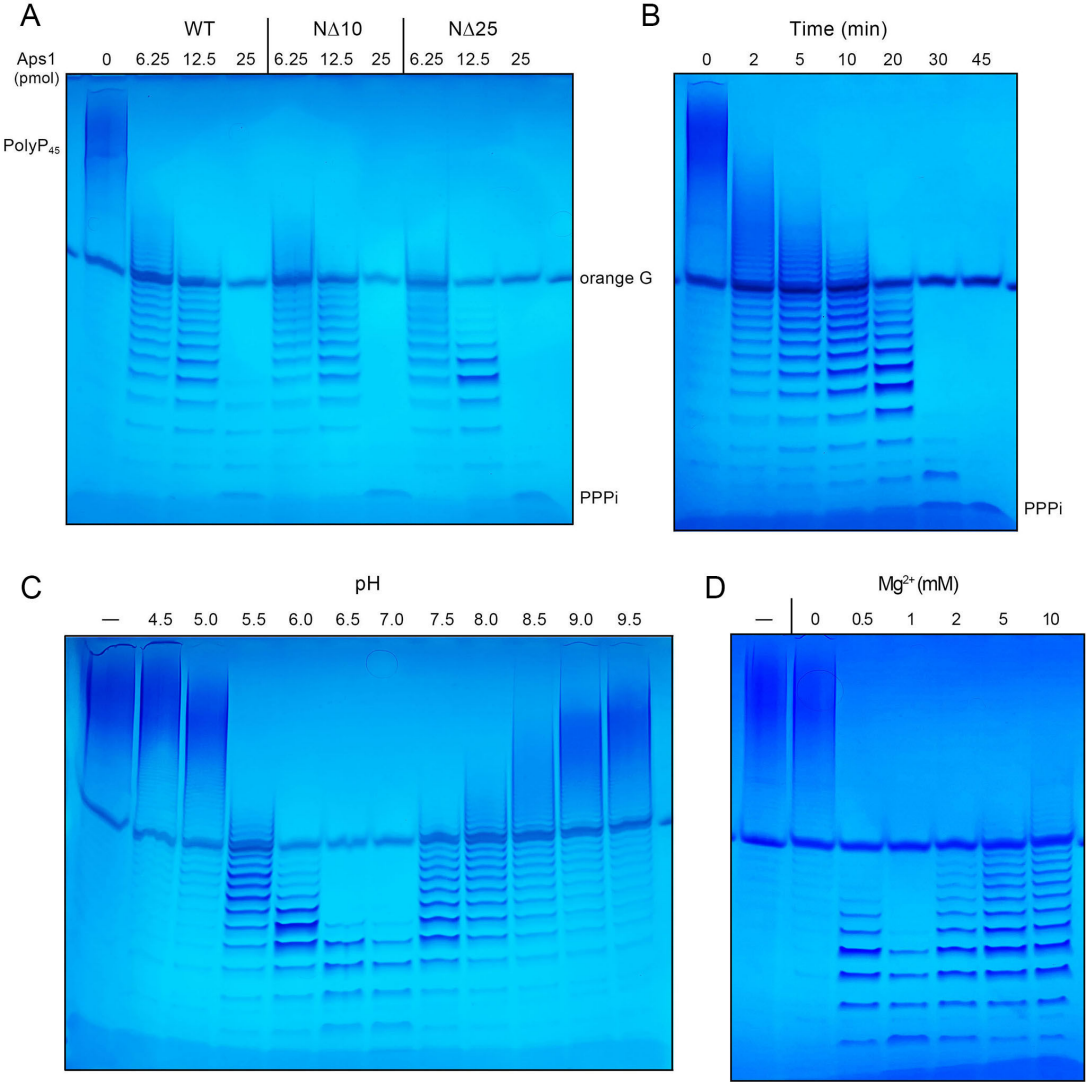
Characterization of the Aps1 inorganic poly-Pase activity. (**A**) Enzyme titration. Reaction mixtures (10 µL) containing 50 mM Tris-HCl (pH 7.4), 5 mM MgCl_2_, 0.2 mM (2 nmol) poly-P_45_, and full-length Aps1, N∆10, or N∆25 as specified were incubated at 37°C for 30 minutes and then quenched by adjustment to 50 mM EDTA. (**B**) Time course. A reaction mixture (80 µL) containing 50 mM Tris-HCl (pH 7.4), 5 mM MgCl_2_, 0.2 mM (16 nmol) poly-P_45_, and 25 pmol full-length Aps1 was incubated at 37°C. At the times specified, aliquots (10  µL) were withdrawn and quenched immediately by adjustment to 50  mM EDTA. (**C**) pH profile. Reaction mixtures (10 µL) containing 50 mM buffer (either Tris-acetate pH 4.5, 5.0, 5.5, 6.0, and 6.5 or Tris-HCl pH 7.0, 7.5, 8.0, 8.5, 9.0, and 9.5), 5 mM MgCl_2_, 0.2 mM (2 nmol) poly-P_45_, and 25 pmol full-length Aps1 were incubated at 37°C for 20 minutes and then quenched with EDTA. (**D**) Magnesium dependence. Reaction mixtures (10 µL) containing 50 mM Tris-HCl (pH 7.0), 0.2 mM poly-P_45_, 12.5 pmol full-length Aps1, and either 0, 0.5, 1, 2, 5, or 10 mM MgCl_2_ as specified were incubated at 37°C for 20 minutes. A control mixture lacking enzyme is shown in lane –. In panels A–D, the reaction products were analyzed by 36% PAGE, and the polyphosphate compounds were detected by staining the gel with toluidine blue. The position of a tripolyphosphate standard (Sigma) analyzed in parallel is denoted by PPPi.

To interrogate Aps1 enzymatic function, we reacted full-length Aps1 and the N∆ truncations with 5 mM MgCl_2_ and 0.2 mM inorganic polyphosphate (poly-P_45_) with an average linear polymer chain length of 45. The products were analyzed by electrophoresis through a 36% polyacrylamide gel, and the polyphosphate chains were visualized by staining the gel with toluidine blue. All three recombinant proteins elicited an Aps1 concentration-dependent conversion of poly-P_45_ to a ladder of progressively shorter poly-P species, culminating in the production of tripolyphosphate (PPP_i_) as the apparent end-product (Fig. 3A). This initial experiment established that (i) fission yeast Aps1, like its budding yeast Ddp1 and human DDIP homologs, has poly-P endopolyphosphatase activity and (ii) the N-terminal 25-aa peptide is dispensable for catalysis. All subsequent characterizations were performed with full-length Aps1.

### Characterization of the Aps1 inorganic polyphosphatase

**Fig 4:**
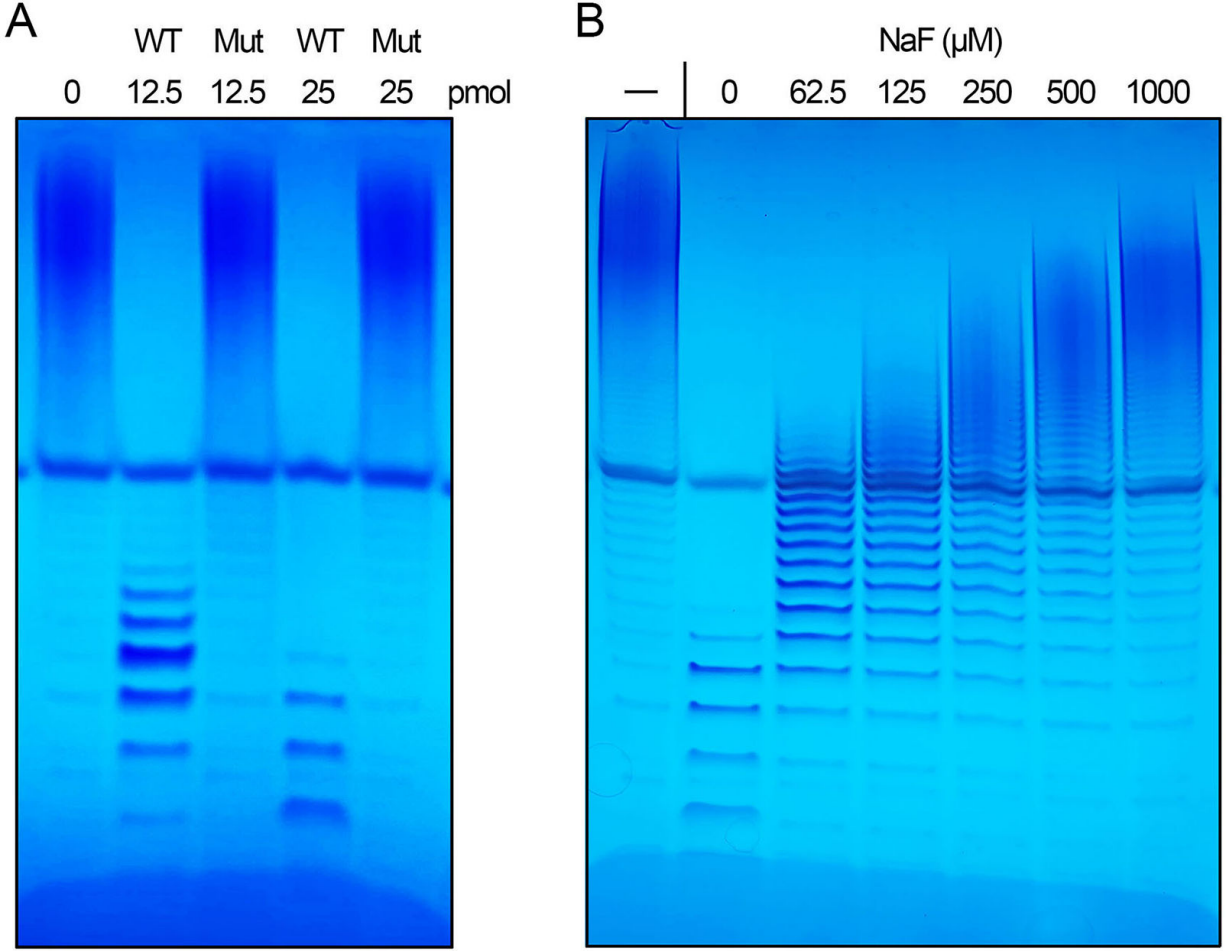
Aps1 poly-Pase activity is abolished by E89A–E93A mutation and inhibited by fluoride. (**A**) Reaction mixtures (10 µL) containing 50 mM Tris-HCl (pH 7.0), 1 mM MgCl_2_, 0.2 mM poly-P_45_, and 12.5 pmol or 25 pmol of Aps1 (WT) or Aps1-(E89A-E93A) (Mut) were incubated at 37°C for 20 minutes. (**B**) Reaction mixtures (10 µL) containing 50 mM Tris-HCl (pH 7.0), 1 mM MgCl_2_, 0.2 mM poly-P_45_, 12.5 pmol Aps1, and NaF as specified were incubated at 37°C for 20 minutes. The reaction products were analyzed by 36% PAGE and detected by toluidine blue staining.

The temporal profile of the inorganic poly-Pase reaction underscores that Aps1 progressively shortened the polyphosphate chains, until virtually all of the input substrate was converted to tri- and tetrapolyphosphate (Fig. 3B). Poly-Pase activity was optimal at pH 6.5 to 7.0 and declined steadily as the pH was reduced to 5.0 or increased to 9.0 (Fig. 3C). Poly-P hydrolysis depended on exogenous magnesium and was optimal at 1 mM MgCl_2_ (Fig. 3D). To affirm that the observed poly-Pase activity is inherent to the recombinant Aps1 protein, we produced and purified a full-length mutant, Aps1-(E89A–E93A), in which two of the essential metal-binding glutamates of the Aps1 Nudix motif (17) were changed to alanine. The Aps1-(E89A–E93A) mutant failed to hydrolyze poly-P_45_ when assayed in parallel with wild-type Aps1 (Fig. 4A). Lonetti *et al*. (18) reported that the poly-Pase activity of *S. cerevisiae* Ddp1 was inhibited by µM concentrations of sodium fluoride. In this study, we found that µM levels of sodium fluoride inhibited poly-P hydrolysis by Aps1 (Fig. 4B).

### Aps1 hydrolysis of inositol pyrophosphates

**Fig 5:**
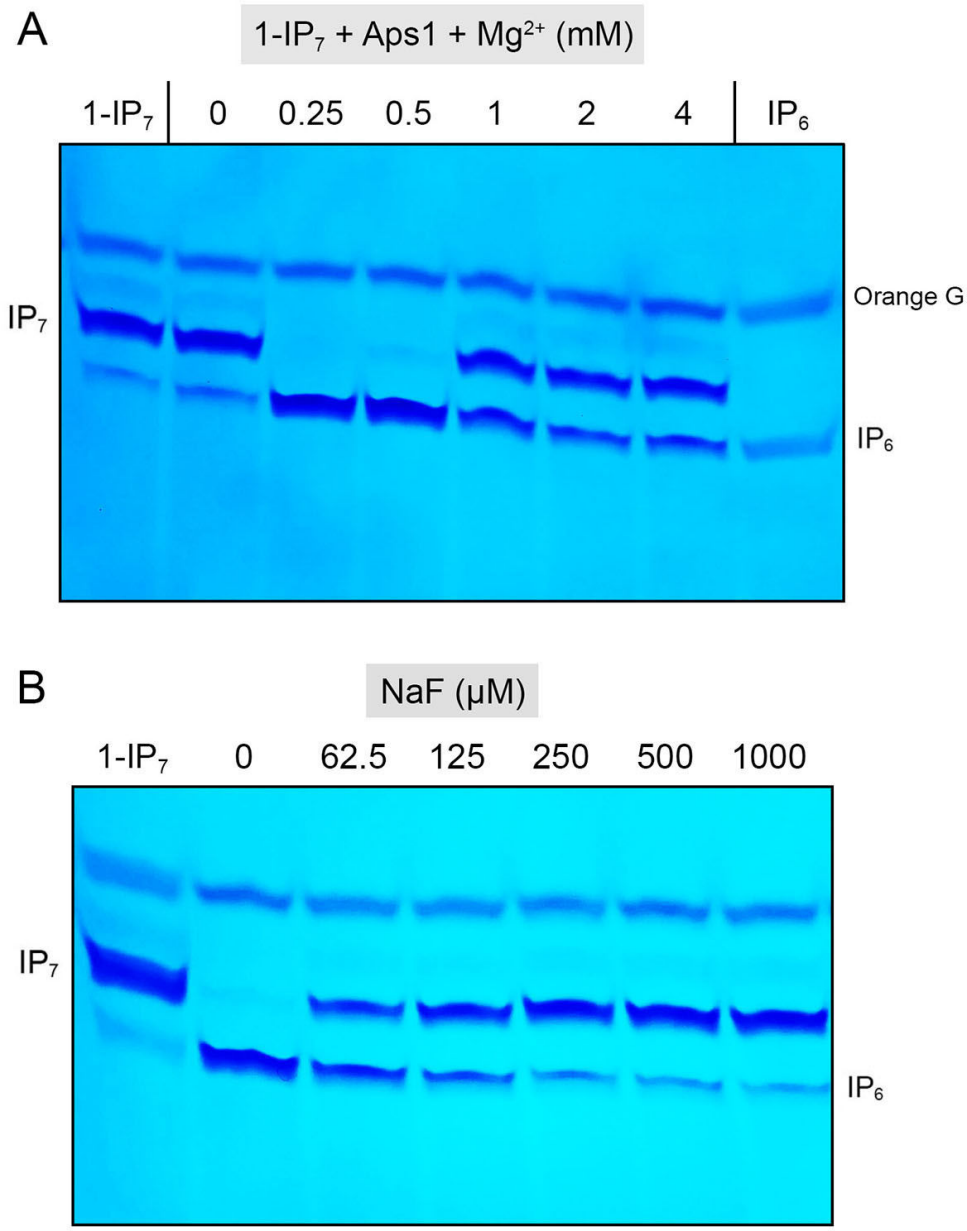
Magnesium-dependent hydrolysis of 1-IP_7_ and inhibition by fluoride. (**A**) Reaction mixtures (10 µL) containing 50 mM Tris-HCl (pH 7.0), 0.25 mM 1-IP_7_, 100 pmol Aps1, and MgCl_2_ as specified were incubated at 37°C for 30 minutes. (**B**) Reaction mixtures (10 µL) containing 50 mM Tris-HCl (pH 7.0), 0.25 mM MgCl_2_, 0.25 mM 1-IP_7_, 20 pmol Aps1, and NaF as specified were incubated at 37°C for 30 minutes. The reaction products were analyzed by 36% PAGE and detected by toluidine blue staining.

Aps1 (10 µM) was reacted for 30 minutes with 0.25 mM 1-IP_7,_ and varying concentrations of magnesium and the products were analyzed by electrophoresis through a 36% polyacrylamide gel. The phosphorylated species were visualized by staining the gel with toluidine blue (Fig. 5A). Aps1 effected quantitative conversion of 1-IP_7_ to IP_6_ when the Mg^2+^ concentration (0.25 mM) was equivalent to that of the 1-IP_7_ substrate. Activity was maintained at 0.5 mM Mg^2+^, but was significantly inhibited at 1, 2, and 4 mM Mg^2+^ (Fig. 5A). The inositol pyrophosphatase activity of mammalian DIPP proteins is inhibited by fluoride (13, 16). In this study, we found that Aps1 hydrolysis of 1-IP_7_ is sensitive to fluoride inhibition (Fig. 5B).

**Fig 6:**
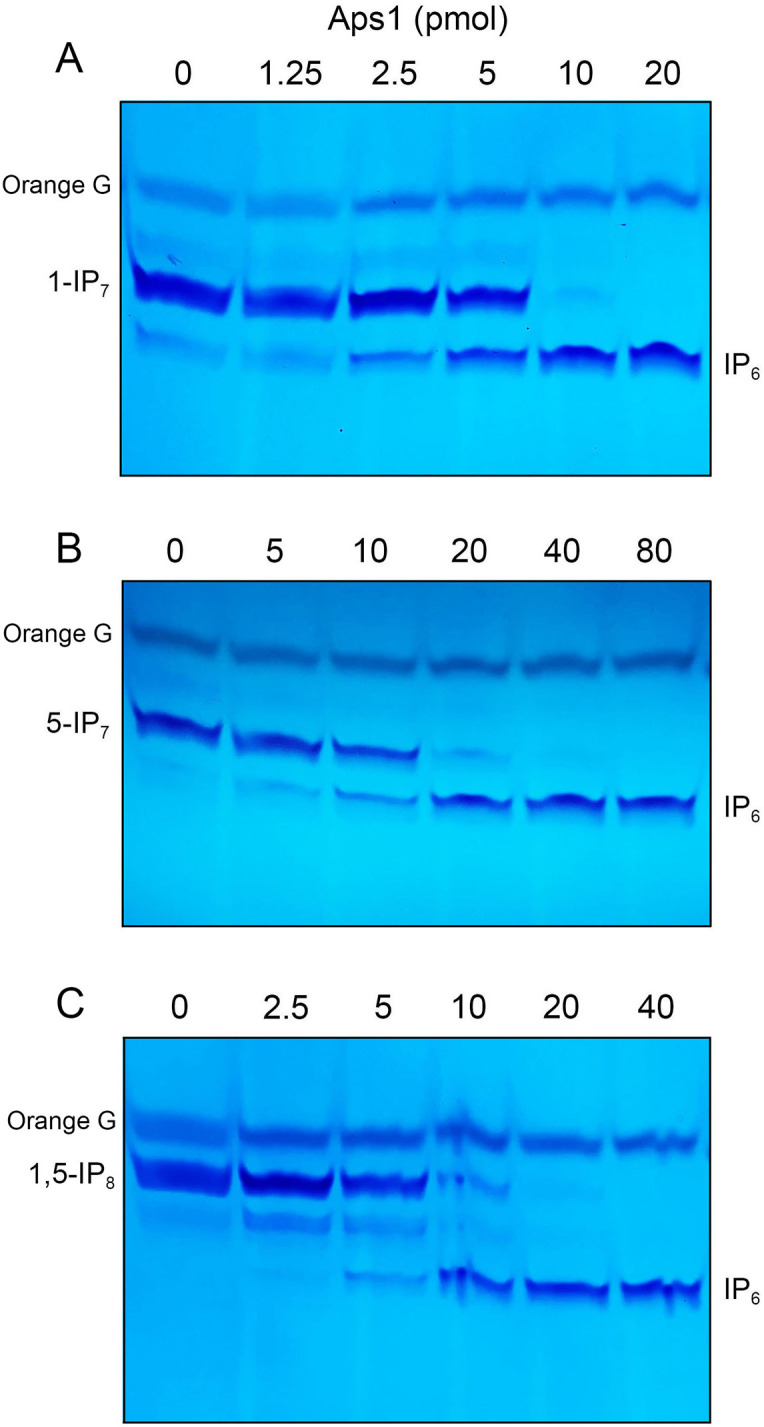
Hydrolysis of 1-IP_7_, 5-IP_7_, and 1,5-IP_8_. Reaction mixtures (10 µL) containing 50 mM Tris-HCl (pH 7.0), 0.25 mM MgCl_2_, 0.25 mM 1-IP_7_ (panel A), 0.25 mM 5-IP_7_ (panel B), 0.25 mM 1,5-IP_8_ (panel C), and Aps1 as specified were incubated at 37°C for 30 minutes. The reaction products were analyzed by 36% PAGE and detected by toluidine blue staining.

**Fig 7:**
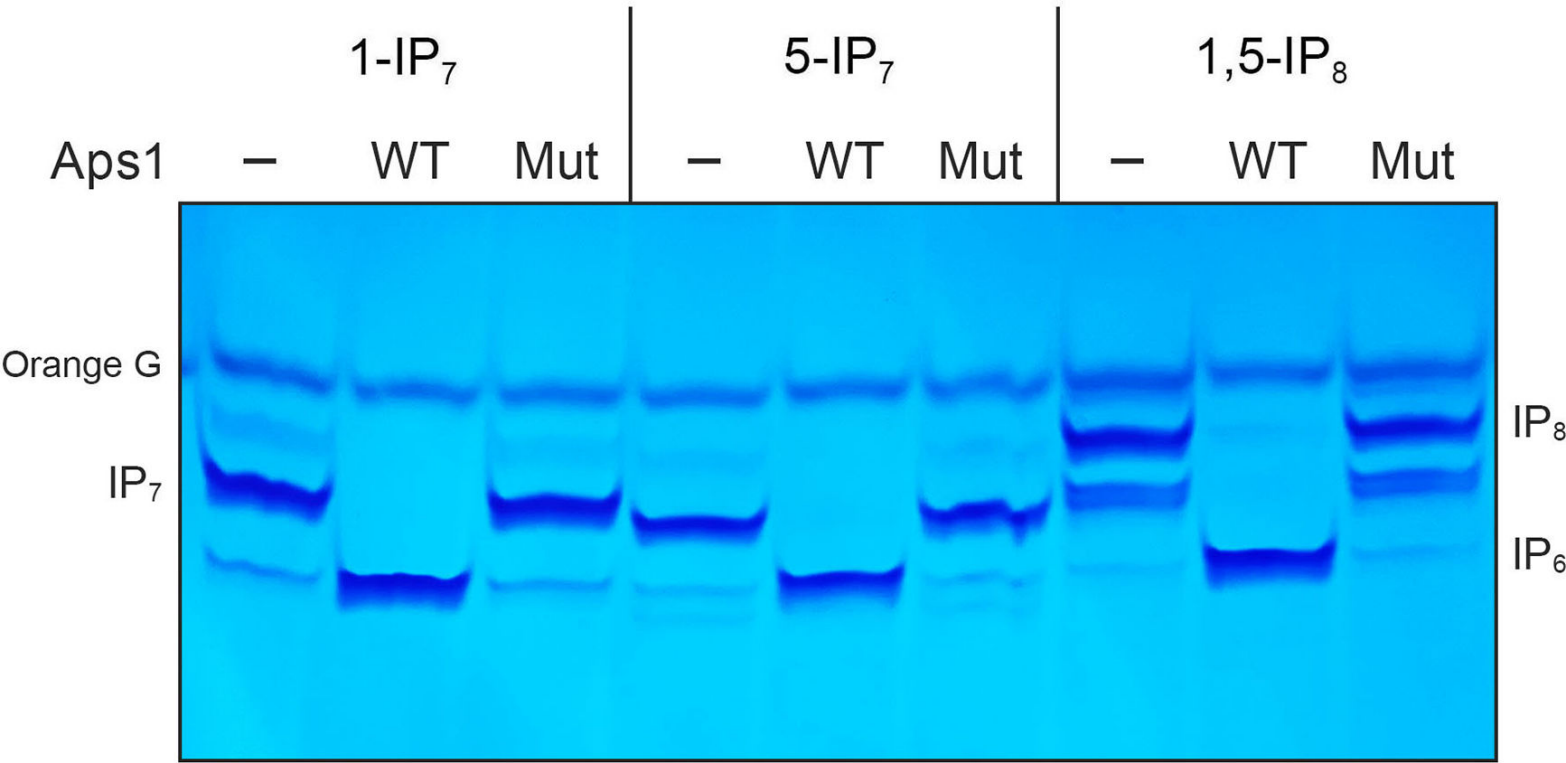
E89A–E93A mutation abolishes inositol pyrophosphatase activity. Reaction mixtures (10 µL) containing 50 mM Tris-HCl (pH 7.0), 0.25 mM MgCl_2_, 0.25 mM 1-IP_7_ , 5-IP_7_, 1,5-IP_8_, and 100 pmol of Aps1 (WT) or Aps1-(E89A-E93A) (Mut) were incubated at 37°C for 30 minutes. Aps1 was omitted from control reactions in lanes –. The reaction products were analyzed by 36% PAGE and detected by toluidine blue staining.

The extent of hydrolysis of 1-IP_7_ to IP_6_ in 0.25 mM Mg^2+^ was proportional to input Aps1 (Fig. 6A). Based on the observation that 10 pmol of Aps1 sufficed to convert virtually all the input 1-IP_7_ (2.5 nmol) to IP_6_, we estimated a turnover number of ~8.3 min^−1^. Parallel titrations performed with 0.25 mM 5-IP_7_ as the substrate showed that Aps1 specific activity with 5-IP_7_ was approximately half of the activity observed with 1-IP_7_ (Fig. 6B). Aps1 converted 1,5-IP_8_ to IP_6_ with specific activity similar to that with 1-IP_7_ (Fig. 6C). These activities are comparable to the *k*_cat_ value of 10.2 min^−1^ reported by Safrany *et al*. (14) for Aps1 hydrolysis of 5-IP_7_. The Aps1-(E89A-E93A) mutant failed to hydrolyze 1-IP_7_, 5-IP_7_, and 1,5-IP_8_ when assayed in parallel with wild-type Aps1, which sufficed to convert all of the input substrate to IP_6_ (Fig. 7).

### Aps1 genetic interaction with Siw14

**Fig 8:**
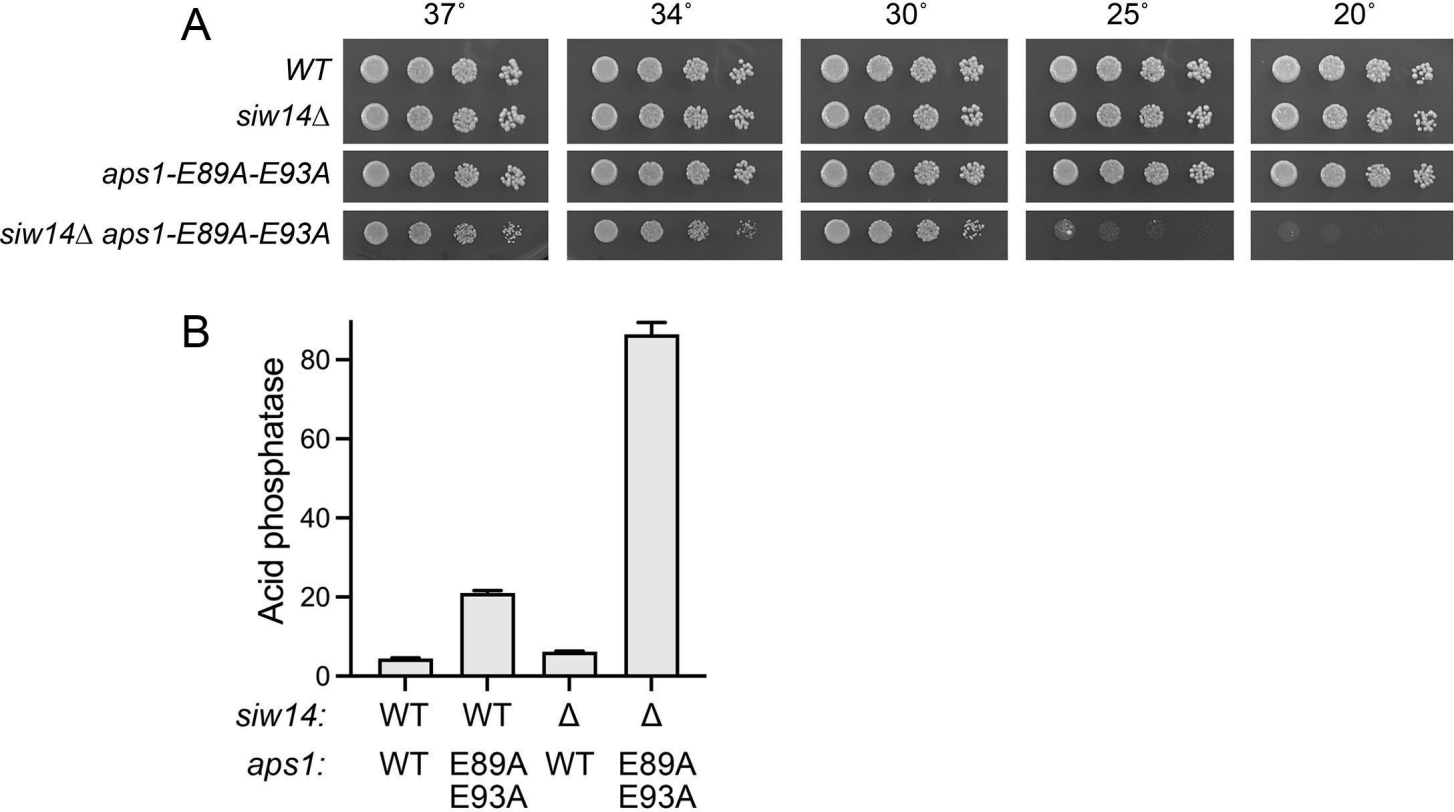
Aps1 mutation E89A–E93A does not result in synthetic lethality with *siw14*∆. (**A**) Serial fivefold dilutions of *S. pombe* wild-type cells and cells bearing the indicated *siw14* and *aps1* alleles were spot-tested for growth on YES agar at the temperatures specified. (**B**) The indicated strains were grown to *A*_600_ of 0.5 to 0.8 in liquid culture in YES medium at 30°C. Cells were then harvested, washed with water, and assayed for Pho1 acid phosphatase activity by conversion of *p*-nitrophenylphosphate to *p*-nitrophenol. Activity is expressed as the ratio of *A*_410_ (*p*-nitrophenol production) to *A*_600_ (input cells).

**Fig 9:**
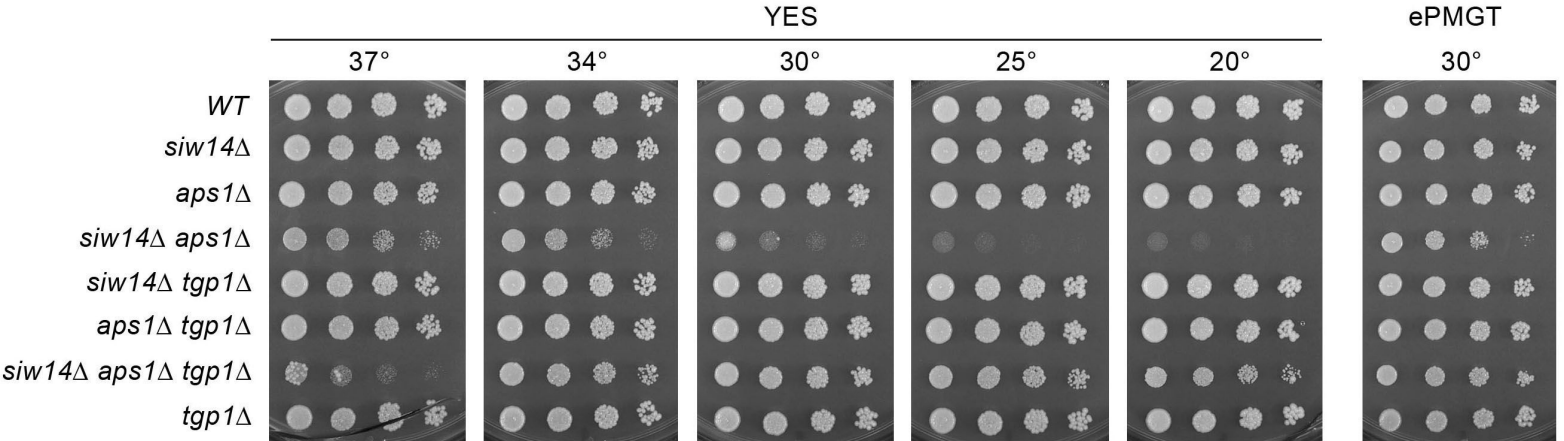
Synthetic growth defect of *siw14*∆ *aps1*∆ is rescued by *tgp1*∆. Serial fivefold dilutions of fission yeast strains (as specified on the left) were spot-tested for growth on YES agar and ePMGT agar at the indicated temperatures.

**Fig 10:**
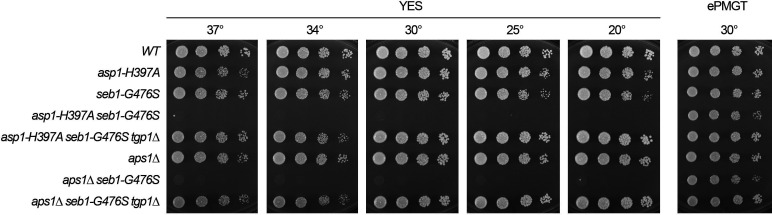
Synthetic growth defect of *aps1*∆ *seb1-G476S* and *asp1-H397A seb1-G476S* is suppressed by deletion of *tgp1*. Serial fivefold dilutions of *S. pombe* wild-type, single-mutant, and double-mutant strains with the indicated *asp1*, *seb1*, *aps1,* and *tgp1* alleles were spot-tested for growth on YES agar and ePMGT agar at the temperatures specified.

None of the three known fission yeast inositol pyrophosphatase activities is essential *per se* for vegetative growth; i.e., the pyrophosphatase-defective *asp1-H397A* strain and the *aps1*∆ and *siw14*∆ null strains grow on YES agar at 20°C to 37°C (Fig. 8 to 10). However, *asp1-H397A* is synthetically lethal with *aps1*∆ (26), suggesting that simultaneous ablation of these two pyrophosphatases results in accumulation of toxic levels of 1,5-IP_8_. Moreover, we found that *aps1*∆ was synthetically lethal with *siw14*∆, as gauged by the inability to recover viable *aps1*∆ *siw14*∆ haploid progeny of a pairwise cross after plating a large population of spores on YES medium, thus signifying that Aps1 and Siw14 pyrophosphatases have essential but redundant functions in fission yeast (12, 30). Multiple lines of genetic evidence indicate that the lethality of the *aps1*∆ *siw14*∆ strain arises from unconstrained precocious transcription termination caused by too much IP_8_. That is to say, (i) the synthetic lethality of *siw14*∆ *aps1*∆ depends on the synthesis of 1,5-IP_8_ by the Asp1 kinase and (ii) *siw14*∆ *aps1*∆ lethality is suppressed by loss-of-function mutations of multiple components of the fission yeast 3'-processing/termination machinery, including CPF (cleavage and polyadenylation factor) subunits Ctf1, Dis2, Ppn1, Swd22, and Ssu72 and termination factor Rhn1 (12). Previously, we showed that Siw14 pyrophosphatase activity is pertinent to the synthetic lethality of *siw14*∆ with *aps1*∆, i.e., the catalytically inert *siw14-C189S* active site mutant phenocopied *siw14*∆ with respect to synthetic lethality with *aps1*∆ (12).

The issue is whether the catalytic activity of Aps1 is necessary for viability in the *siw14*∆ background. To address this question, we constructed a *kanMX*-marked *aps1-(E89A–E93A*) mutant strain of fission yeast encoding a catalytically inactive Aps1 protein. *aps1-(E89A-E93A*) cells grew as well as wild-type cells on YES agar (Fig. 8A) and displayed a modest derepression of Pho1 expression (Fig. 8B), akin to that seen for *aps1*∆ cells (26). A genetic cross of *aps1-(E89A–E93A*) and *siw14*∆ strains followed by random spore analysis (35) and selection for the *kanR*- and *hygR*-marked *aps1-(E89A–E93A*) and *siw14*∆ loci yielded double-mutant progeny that germinated and formed colonies on YES agar. After amplification in liquid YES medium at 30°C and spotting serial dilutions of wild-type, single-mutant, and double-mutant cells on YES agar, we found that the *aps1-(E89A-E93A) siw14*∆ strain grew slowly at 30°C, 34°C, and 37°C, as gauged by colony size, and failed to form macroscopic colonies at 25°C or 20°C (Fig. 8A). We conclude that the catalytically dead Aps1-(E89A–E93A) mutant retains partial biological activity in complementing the inviability of *siw14*∆ *aps1*∆ spores. Pho1 acid phosphatase activity in the *aps1-(E89A–E93A) siw14*∆ strain was fourfold higher than that of the *aps1-(E89A–E93A*) single mutant (Fig. 8B), consistent with an additive effect on *pho1* expression of ablating the functionally redundant Siw14 and Aps1 pyrophosphatases.

### *tgp1*^+^ deletion suppresses the synthetic growth defect of *siw14*∆ *aps1*∆

Various *asp1* mutations that delete or inactivate the Asp1 pyrophosphatase domain elicit growth defects in YES medium, ranging from severe sickness to lethality. The lethal alleles *asp1-STF6* and *asp1-STF9* were found to be defective for outgrowth after spore germination on YES medium (31). A key observation was that the growth defects of these and other *asp1-STF* pyrophosphatase-defective mutants were manifest in medium containing yeast extract but not in a synthetic medium ePMGT. A suppressor screen revealed that IP_8_ toxicosis of *asp1-STF* mutants is caused by (i) a > 40 fold increase in the expression of the inessential *tgp1* gene encoding a glycerophosphocholine transporter and (ii) the presence of glycerophosphocholine in the growth medium, which recapitulates the toxicity of yeast extract to *asp1-STF* cells (31).

The question here is whether the synthetic phenotype of *siw14*∆ *aps1*∆ on YES agar is similarly connected to derepression of *tgp1*. By plating spores from a cross of *siw14*∆ and *aps1*∆ strains on ePMGT medium, rather than on YES, we were able to recover viable *siw14*∆ *aps1*∆ haploids that grew on ePMGT agar at 30°C, albeit more slowly than a wild-type strain or the *siw14*∆ and *aps1*∆ single mutants, as gauged by colony size (Fig. 9). Spot-testing the *siw14*∆ *aps1*∆ strain for growth on YES agar revealed it to be sicker at 30°C vis-à-vis ePMGT. The *siw14*∆ *aps1*∆ cells did not form macroscopic colonies on YES at 25°C or 20°C, but did yield tiny colonies on YES at 34°C and 37°C (Fig. 9). The key point is that the severe growth defects of *siw14*∆ *aps1*∆ cells on YES agar at 20°C–34°C were suppressed by deletion of *tgp1*, as was the small colony phenotype on ePMGT at 30°C (Fig. 9). Note, however, that *tgp1*∆ did not reverse the *siw14*∆ *aps1*∆ growth defect on YES at 37°C and that *siw14*∆ *aps1*∆ *tgp1*∆ cells were slow growing at 20°C, as gauged by colony size (Fig. 9). We surmise that IP_8_-driven overexpression of Tgp1 in *siw14*∆ *aps1*∆ is responsible for most (but not all) of the growth defects observed.

### *tgp1*^+^ deletion suppresses the synthetic growth defect of *aps1*∆ *seb1-G476S*

A prior genetic screen for relief of transcriptional interference with Pho1 acid phosphatase expression unveiled a mechanism by which lncRNA termination is enhanced via a mutation G476S in the RNA-binding domain of an essential termination factor, Seb1 (32). *seb1-G476S* derepressed the *pho1* and *tgp1* mRNAs. RNA analysis showed that the *tgp1*-interfering *nc-tgp1* lncRNA and the *pho1*-interfering *prt* lncRNA were terminated precociously in *seb1-G476S* cells (32). The *seb1-G476S* allele was found to be synthetically lethal with *aps1*∆; *viz*., (i) we were unable to obtain viable double-mutants after screening a large population of haploid progeny of the genetic cross for growth on YES medium and (ii) wild-type progeny and the differentially marked *seb1-G476S* and *aps1*∆ single-mutants were recovered at the expected frequencies (32). The synthetic lethality of *seb1-G476S* with *aps1*∆ was rescued by CPF/Rhn1 loss-of-function alleles (32). In the present context, it was of interest to establish whether *tgp1* overexpression was causal for the *aps1*∆ *seb1-G476S* synthetic phenotype. By plating spores from a cross of *seb1-G476S* and *aps1*∆ strains on ePMGT medium, we recovered viable *seb1-G476S aps1*∆ haploids that grew on ePMGT agar at 30°C, but were unable to grow on YES agar at any of the temperatures tested (Fig. 10). The lethal growth defect on YES was fully suppressed by *tgp1*∆ (Fig. 10).

### *tgp1*∆ suppresses the synthetic growth defect of *asp1-H397A seb1-G476S*

The Asp1 pyrophosphatase mutation *H397A* elicits derepression of the *PHO* genes (26) but does not adversely affect cell growth on YES agar (Fig. 10). We noted previously that combining *asp1-H397A* and *seb1-G476S* exerted a severe (virtually lethal) growth defect on YES agar. In this study, we reconstructed the *asp1-H397A seb1-G476S* double-mutant and found that it grew well on ePMGT at 30°C but failed to grow on YES agar at any temperature (Fig. 10). The lethality of *asp1-H397A seb1-G476S* on YES was fully suppressed by *tgp1*∆ (Fig. 10).

### *tgp1*∆ does not suppresses the lethality of *asp1-H397A aps1∆*

Simultaneous inactivation of the Asp1 and Aps1 pyrophosphatases is lethal; i.e., we were unable to recover an *asp1-H397A aps1*∆ double-mutant after crossing the single-mutant strains and plating spores on YES agar. This synthetic lethality was rescued by loss-of-function mutations in components of the 3’-processing and transcription termination machinery, e.g., CPF subunits Ppn1, Swd22, and Ssu72, and the Pol2 CTD prolyl isomerase Pin1 (26, 36). By contrast, null alleles of CPF subunit Dis2 and termination factor Rhn1 did not rescue the *asp1-H397A aps1*∆ lethality (26). We identified loss-of-function mutations in three SPX domain proteins (Spx1, Gde1, and Vtc4) as suppressors of lethality or sickness associated with *asp1-STF* mutations (29). When these suppressor alleles were tested for their ability to rescue the lethality of *asp1-H397A aps1*∆ in a genetic cross, we found that inactivation of Spx1 allowed for the growth of *asp1-H397A aps1*∆ cells on YES agar at all temperatures, whereas inactivation of Gde1 and Vtc4 did not alleviate the *asp1-H397A aps1*∆ synthetic lethality (29). Recent studies indicated that failure to grow out after germination is the root of the apparent lethality of certain inositol pyrophosphatase mutants (31). We found that we were able to recover a viable but sick *asp1-H397A aps1*∆ *tgp1*∆ triple-mutant haploid in a cross of *asp1-H397A* and *aps1*∆ *tgp1*∆ strains. The *asp1-H397A aps1*∆ *tgp1*∆ cells grew equally poorly at all temperatures on either YES or ePMGT medium (Fig. S1). Thus, we hypothesize that inactivation of the two main inositol pyrophosphatases in fission yeast elevates IP_8_ to a very high level, which elicits toxic transcription termination events independent of the precocious lncRNA termination that leads to Tgp1 overexpression and deleterious uptake of GPC.

### Effects of Aps1, Siw14, and Asp1 mutations on cellular inositol polyphosphate levels

**Fig 11:**
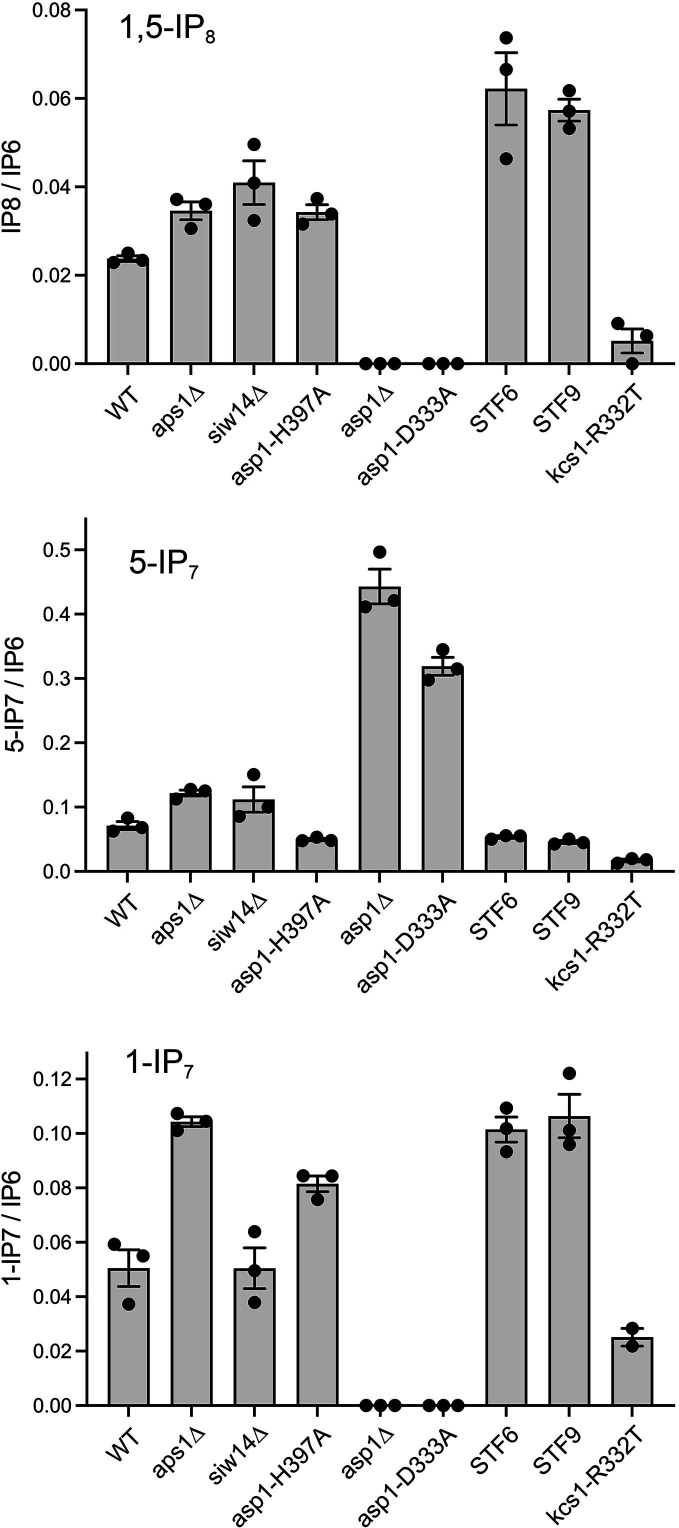
Mutational effects on inositol pyrophosphate levels as gauged by CE-ESI-MS. The inositol pyrophosphate levels in extracts of the indicated fission yeast strains, normalized to that of IP_6_, are shown for 1,5-IP_8_ (top panel), 5-IP_7_ (middle panel), and 1-IP_7_ (bottom panel). The bar heights depict the mean of three individual biological replicates (denoted by dots) ±SEM for all analyses, except for *kcs1-R332T* in the bottom panel, which shows the mean of two individual biological replicates ± the range of values.

The advent of sensitive capillary electrophoresis electrospray ionization mass spectrometry (CE-ESI-MS) methods to profile the pool of cellular IP_6_, 5-IP_7_, 1-IP_7_, and 1,5-IP_8_ (33, 34) enables us to gauge how mutations of enzymes that synthesize and catabolize these molecules affect inositol pyrophosphate dynamics *in vivo*. Whole-cell perchloric acid extracts from 10 *A*_600_ units of wild-type and mutant fission yeast cells growing logarithmically in ePMGT medium were adsorbed to titanium dioxide beads to enrich for inositol polyphosphates, which were eluted with 3% ammonium hydroxide. CE-ESI-MS was performed as described (33) on three biological replicates (independent cultures) of each fission yeast strain. The amounts (in pmol) of IP_6_, 5-IP_7_, 1-IP_7_, and 1,5-IP_8_ in each sample were determined, and the levels of 5-IP_7_, 1-IP_7_, and 1,5-IP_8_ were normalized to that of IP_6_ to correct for any variations in sample recovery during extractions and TiO_2_ bead affinity enrichment. The data for 1,5-IP_8_, 5-IP_7_, and 1-IP_7_ are plotted in Fig. 11. In wild-type fission yeast, the 1,5-IP_8_/IP_6_, 5-IP_7_/IP_6_, and 1-IP_7_/IP_6_ ratios were 0.0238, 0.0713, and 0.0505, respectively.

The results obtained for the various fission yeast mutants affirm and extend those of earlier studies that relied on metabolic labeling of cells with ^3^H-inositol and fractionation of ^3^H-labeled inositol polyphosphates by ion exchange chromatography (3, 4). The key conclusions are listed below:

1. The Asp1 kinase, which is specific for phosphorylation of the 1-phosphate position *in vitro*, is uniquely responsible for synthesis of 1,5-IP_8_ and 1-IP_7_, neither of which was detected in *asp1*∆ cells lacking Asp1 protein or in *asp1-D333A* cells expressing a kinase-dead Asp1 enzyme.
2. Absence of Asp1 kinase leads to accumulation of high levels of 5-IP_7_ (*asp1*∆/WT = 6.2; *P* value 0.0037).
3. Absence of the Aps1 Nudix-type pyrophosphatase, which removes either the 1 or 5 β-phosphate *in vitro*, leads to a modest increase in 1,5-IP_8_ (*aps1*∆/WT = 1.46; *P* value 0.025) and 5-IP_7_ (*aps1*∆/WT = 1.71; *P* value 0.0036) but has a bigger effect on accumulation of 1-IP_7_ (*aps1*∆/WT = 2.06; *P* value 0.011). This suggests that Aps1 might have a preference for dephosphorylating 1-IP_7_ *in vivo*. These *in vivo* data resonate with our finding that Aps1 specific activity with 1-IP_7_ was approximately twice that of the activity seen with 5-IP_7_.
4. Absence of the Siw14 cysteinyl-phosphate-type pyrophosphatase, which removes either the 1 or 5 β-phosphate *in vitro* (26), leads to modest increases in 1,5-IP_8_ (*siw14*∆/WT = 1.72; *P* value 0.072) and 5-IP_7_ (*siw14*∆/WT = 1.57; *P* value 0.16) but has no effect on accumulation of 1-IP_7_ (*siw14*∆/WT = 1.0). This suggests that Siw14 prefers to hydrolyze the 5 β-phosphate *in vivo*. We noted previously that Siw14 was approximately twofold more active at hydrolyzing 5-IP_7_ versus 1-IP_7_ when *in vitro* enzyme titrations were performed in the presence of 1 mM magnesium (26).
5. We gained insights into how different mutations in the histidine phosphatase-type pyrophosphatase domain of Asp1 have a gradient of effects on fission yeast growth in YES medium, ranging from benign (H397A) to toxic (STF6 and STF9). By growing the mutants in ePMGT medium permissive for growth of *asp1-STF* strains, the toxicosis (or lack thereof) appears to correlate with the degree of accumulation of 1,5-IP_8_ in the mutant versus wild-type. To wit, the 1,5-IP_8_ mutant/WT ratios were 1.44 for *asp1-H397A* (*P* value 0.016), 2.62 for *asp1-STF6* (*P* value 0.042), and 2.41 for *asp1-STF9* (*P* value 0.0035).
6. Kcs1 is the kinase that converts IP_6_ to 5-IP_7_. Kcs1 is essential for viability. We recovered the *kcs1-R332T* allele in a screen for suppression of the toxicity of *asp1-STF* mutants and proposed that the R332T mutation reduces IP_6_ kinase activity and thereby limits the pool of cellular 5-IP_7_, which is the substrate for synthesis of 1,5-IP_8_ by Asp1 kinase (31). The CE-ESI-MS results support this view, insofar as 1,5-IP_8_ and 5-IP_7_ levels are reduced in *kcs1-R332T* cells, both in “raw” levels and when normalized to IP_6_. The normalized *kcs1-R332T*/WT ratios are as follows: 1,5-IP_8_ (0.21; *P* value 0.016) and 5-IP_7_ (0.24; *P* value 0.007).

## DISCUSSION

The results herein extend our knowledge of the biochemical activities and genetic interactions of the fission yeast Nudix pyrophosphatase Aps1. With respect to biochemistry, we find that (i) Aps1 is a magnesium-dependent endopolyphosphatase that converts linear poly-P into shorter oligo-P species, culminating in tripolyphosphate as an apparent end-product; (ii) Aps1 is an inositol pyrophosphatase that hydrolyzes the β-phosphates of 5-IP_7_, 1-IP_7_, and 1,5-IP_8_ to yield IP_6_ as an end-product; (iii) Aps1 displays a ~twofold preference for hydrolysis of 1-IP_7_ versus 5-IP_7_; (iv) Aps1 endopolyphosphatase and inositol pyrophosphatase activities are inhibited by fluoride and abolished by alanine mutations of the conserved Nudix-box glutamates; and (v) the N-terminal 25-aa segment of Aps1 is dispensable for catalysis. The demonstration of Aps1’s endopolyphosphatase activity *in vitro* resonates with our previous finding that the polyphosphate content of an *aps1*∆ strain, as gauged by PAGE and toluidine blue staining, was slightly higher than that of an *aps1*^+^ wild-type strain, especially the population of shorter polymers running at the same rate or faster than the Orange G dye (29). These results suggest that Aps1 contributes to polyphosphate catabolism *in vivo*.

The genetic and CE-ESI-MS analyses presented here clarify the contributions of Aps1 and two other inositol pyrophosphatases (Siw14 and Asp1) to IP_8_ agonism of precocious transcription termination of the lncRNAs that interfere with the mRNA promoters of the *PHO* regulon genes (21), which can result in cytotoxicity if severe (31). We affirm that the Asp1 kinase is the sole enzyme responsible for synthesis of 1,5-IP_8_ in fission yeast and that absence of Asp1 kinase leads to overaccumulation of its substrate 5-IP_7_. In contrast, the absence of Aps1 or Siw14 or an H397A mutation of the pyrophosphatase active site of Asp1 elicits modest increases in cellular IP_8_ levels. While mutational ablation of IP_8_ synthesis results in hyper-repression of the *PHO* genes *tgp1*, *pho1*, and *pho84* (26), mutations of the three inositol pyrophosphatases exert disparate effects on gene expression, notwithstanding their similar extents of IP_8_ accumulation (*aps1*∆/WT = 1.46; *siw14*∆/WT = 1.72; *asp1-H397A*/WT = 1.44). To wit, transcriptome profiling of *siw14*∆ cells showed no changes in the *PHO* regulon and identified only two mRNAs that were upregulated and 10 mRNAs that were downregulated (12). Thus, Siw14 *per se* has little impact on gene expression. Transcriptome profiling of *aps1*∆ cells highlighted 19 mRNAs that were upregulated, including *tgp1*, *pho1*, and *pho84* (26). In *asp1-H397A* cells, 65 mRNAs were upregulated, including *tgp1*, *pho1*, and *pho84* (26). The fold derepression of the *PHO* genes was greater in *asp1-H397A* cells than in *aps1*∆ cells. The reasons for this disparate impact are unclear, but it might reflect differential intracellular localization of the fission yeast inositol pyrophosphatases, leading to differential effects of their mutation on IP_8_ levels in discrete cellular compartments. Current knowledge is scant in this regard.

What is clear is that there are profound mutational synergies between the three inositol pyrophosphatases, whereby *aps1*∆ *siw14*∆ and *aps1*∆ *asp1-H397A* double mutations are lethal (12, 26). Recent studies of the *asp1-STF6/9* alleles, in which large segments of the Asp1 C-terminal pyrophosphatase domain are deleted (27), revealed that their severe growth defect on YES medium (or on ePMGT medium supplemented with glycerophosphocholine) was caused by IP_8_-driven derepression of the glycerophosphocholine transporter Tgp1 and could be suppressed by an inactivating *tgp1* mutation or a targeted *tgp1*∆ deletion (31). Thus, it was suggested that a supra-threshold level of IP_8_ can lead to Tgp1-dependent toxicity via the agonist effect of IP_8_ on precocious termination of the *nc-tgp1* lncRNA that interferes with *tgp1* mRNA synthesis (31, 37). Consistent with this threshold model, we find that *asp1-STF* cells accumulate higher levels of IP_8_ than *asp1-H397A* cells.

In this study, we provide evidence that the synthetic lethality of *aps1*∆ *siw14*∆ is also caused by excessive Tgp1-driven uptake of GPC, insofar as we were able to recover a viable *aps1*∆ *siw14*∆ mutant on ePMGT medium (lacking GPC) and to suppress the severe growth defect of *aps1*∆ *siw14*∆ on YES at 30°C by deleting *tgp1*. The *aps1*∆ and *asp1-H397A* mutations are both synthetically lethal with *seb1-G476S,* and this lethality is manifest in YES medium (but not ePMGT) and suppressed by deleting *tgp1*.

A surprising finding was that the catalytically defective *aps1-(E89A–E93A*) allele was not lethal in the *siw14*∆ background, though the *aps1-(E89A–E93A) siw14*∆ strain was slow growing. Our inference is that the Aps1 protein retains some biological activity in the absence of catalysis, which sustains viability of *siw14*∆ cells. We speculate that the Aps1-(E89A–E93A) protein might retain the capacity to bind IP_8_ and thus sequester some of the excess IP_8_ that accumulates when Siw14 and Aps1 pyrophosphatases are missing or crippled. On the flip side, the pyrophosphatase-dead *siw14-C189S* allele cannot sustain the viability of *aps1*∆ cells (12).

The fact that the severe growth defect of *aps1*∆ *asp1-H397A* could not be alleviated either by growth on ePMGT medium or deletion of *tgp1* suggests that IP_8_ levels in this double-pyrophosphatase mutant exceed an even higher threshold beyond which overzealous 3'-processing/termination affecting genes other than *tgp1* results in cytotoxicity. Consistent with this second threshold 3'-processing/termination model, we found that *aps1*∆ *asp1-H397A* lethality was suppressed by the *ssu72-C13S* allele encoding a catalytically inactive version of the Ssu72 CTD phosphatase subunit of CPF (26). Even taking into account that Ssu72 mutation will soften the derepression of the *PHO* genes by *aps1*∆ *asp1-H397A*, an instructive finding was that the steady-state level of *tgp1* mRNA (assayed by primer extension) was much higher in the *aps1*∆ *asp1-H397A* context than in the *asp1-H397A* single mutant (26).

Finally, our CE-ESI-MS results, taken in light of *in vitro* studies of recombinant enzymes, provide clues to the substrate preferences of the fission yeast inositol pyrophosphatases, whereby Aps1 seems to favor hydrolysis of 1-IP_7,_ while Siw14 favors hydrolysis of 5-IP_7_, as gauged by which IP_7_ species accumulates most in the respective null mutants. These preferences are more subtle for the fission yeast enzymes Aps1 and Siw14 vis-à-vis their budding yeast orthologs Ddp1 and Siw14, as revealed by the recent work of Chabert *et al*. (38) who conducted a CE-ESI-MS analysis of inositol pyrophosphates in wild-type and mutant strains of *S. cerevisiae*. The ratios of IP_8_ to 5-IP_7_ to 1-IP_7_ are similar in wild-type *S. pombe* (0.33/1.0/0.7) and wild-type *S. cerevisiae* (0.47/1.0/0.71). A noteworthy distinction between the two species is that while a fission yeast *asp1*∆ strain had no detectable 1,5-IP_8_ or 1-IP_7_, the corresponding budding yeast *vip1*∆ mutant retained 25% of the wild-type concentration of IP_8_ and 10% of the wild-type level of 1-IP_7_, indicating that budding yeast has an alternative kinase capable of phosphorylating the 1-phosphate group of 5-IP_7_ and IP_6_ (38). Most pertinent to the present work is their finding that *ddp1*∆ cells had a tenfold increase in 1-IP_7_ but no change in 5-IP_7_ and only a 20% increase in 1,5-IP_8_, signifying that budding yeast Ddp1 is acting predominantly on 1-IP_7_ *in vivo* (38). Conversely, budding yeast *siw14*∆ cells accumulate fivefold higher levels of 5-IP_7_ versus wild-type, a 25% increase in 1,5-IP_8_, and no significant change in 1-IP_7_ (38). The latter result resonates with biochemical evidence that purified *S. cerevisiae* Siw14 has vigorous activity in hydrolyzing the 5-β-phosphate of 5-IP_7_ and 1,5-IP_8_ but negligible activity against 1-IP_7_ (10).

## MATERIALS AND METHODS

### Recombinant *S. pombe* Aps1

The ORF encoding full-length Aps1 was PCR-amplified from *S. pombe* complementary DNA (cDNA) with primers that introduced a BamHI site immediately flanking the start codon and an XhoI site downstream of the stop codon. Two Aps1 N-terminal truncations—N∆10 and N∆25—were generated by PCR using forward primers that introduced a BamHI site preceding codons 11 and 26. The PCR products were digested with BamHI and XhoI and inserted between the BamHI and XhoI sites of pET28b-His_10_Smt3 to generate T7 RNA polymerase–based expression plasmids encoding the full-length Aps1, N∆10, or N∆25 polypeptides fused to an N-terminal His_10_Smt3 tag. A double-alanine mutation, E89A–E93A, was introduced into the full-length Aps1 expression plasmid by two-stage overlap extension PCR with mutagenic primers. All plasmid inserts were sequenced to exclude the presence of unwanted mutations. The pET28b-His_10_Smt3-Aps1 plasmids were transfected into *E. coli* BL21(DE3) cells. Cultures (1,000 mL) amplified from single kanamycin-resistant transformants were grown at 37°C in Luria–Bertani medium containing 50 µg/mL kanamycin until the *A*_600_ reached 0.6 to 0.7. The cultures were chilled on ice for 1 hour, adjusted to 2.2% (v/v) ethanol and 0.5 mM IPTG, and then incubated for 18 hours at 17°C with constant shaking. Cells were harvested by centrifugation and stored at −80°C. All subsequent steps were performed at 4°C. Cells were thawed and resuspended in 25 mL of buffer A (50 mM Tris–HCl, pH 7.4, 500 mM NaCl, 20 mM imidazole, and 10% glycerol), containing one complete EDTA-free protease inhibitor cocktail tablet (Roche). Lysozyme was added to a concentration of 1 mg/mL. After incubation for 30 minutes, the lysate was sonicated, and the insoluble material was removed by centrifugation at 16,000 rpm for 30 minutes. The supernatant was mixed for 1 hour with 5 mL of Ni–nitrilotriacetic acid agarose resin (Qiagen) that had been equilibrated with buffer A. The resin was recovered by centrifugation and washed twice with 50 mL of buffer A. The resin was centrifuged again, resuspended in 20 mL of buffer A, and poured into a column. After washing the column with 20 mL of buffer A, the bound material was eluted with 10 mL of buffer A containing 300 mM imidazole, while collecting 5-mL fractions. The polypeptide compositions of the flow-through and eluate fractions were monitored by SDS-PAGE. The 300 mM imidazole eluate fractions containing His_10_Smt3-Aps1 were supplemented with Smt3-specific protease Ulp1 (Ulp1/His_10_-Smt3-Aps1 ratio of 1:425 [w/w]) and then dialyzed overnight against 2,000 mL of buffer B (50 mM Tris–HCl, pH 7.4, 250 mM NaCl, 20 mM imidazole, 1 mM DTT, and 10% glycerol) containing 1 mM EDTA, during which time the His_10_Smt3 was cleaved. The dialysates were mixed for 1 hour with 3 mL of Ni-nitrilotriacetic acid agarose resin that had been equilibrated with buffer B without EDTA. Tag-free Aps1 proteins were recovered in the flow-through fractions. The Aps1 protein preparations were concentrated by centrifugal ultrafiltration (Amicon Ultra-15; 10 kDa cutoff) to 5 mL volume and then gel-filtered through a 125 mL 16/60 HiLoad Superdex 200 column (GE Healthcare) equilibrated in buffer C (20 mM Tris–HCl, pH 7.4, 150 mM NaCl, 1 mM DTT, 1 mM EDTA, and 5% glycerol) at a flow rate of 0.5 mL/min while collecting 2-mL fractions. The peak fractions were pooled, concentrated by centrifugal ultrafiltration (Amicon Ultra-15; 10 kDa cutoff), and stored at –80°C. Protein concentrations were determined with Bio-Rad dye reagent using bovine serum albumin as the standard. The yields of full-length Aps1, N∆10, N∆25, and Aps1-(E89A–E93A) were 14, 18, 15, and 14.5 mg per liter of bacterial culture, respectively.

### Inorganic polyphosphatase assay

Reaction mixtures (10 µL) containing 50 mM Tris-HCl, pH 7.4 or 7.0 (as specified), 0.2 mM poly-P45 (Sigma, Cat # S4379-500MG, Lot # SLBX2788), and MgCl_2_ and Aps1 protein (WT or variants) as specified in the figure legends were incubated for 20 or 30 minutes at 37°C. Reactions were terminated by adjustment to 50 mM EDTA and then mixed with an equal volume of 2 x Orange G loading buffer (10 mM Tris-HCl, pH 7.0, 1 mM EDTA, 30% glycerol, and 0.05% Orange G). The products were analyzed by electrophoresis at 4°C through a 20 cm 36% polyacrylamide gel containing 80 mM Tris-borate (pH 8.3), 1 mM EDTA for 2.5 hours at 10 W constant power. The gel was washed briefly with water and then stained with a solution of 0.1% toluidine blue (Sigma), 20% methanol, 2% glycerol, followed by destaining in 20% methanol.

### Inositol pyrophosphatase assay

Reaction mixtures (10 µL) containing 50 mM Tris-HCl, pH 7.0, 0.25 mM inositol pyrophosphates 1-IP_7_, 5-IP_7_, or 1,5-IP_8_ (chemically synthesized as described; 39–41), 0.25 mM MgCl_2_, and Aps1 protein as specified in the figure legends were incubated for 30 minutes at 37°C. The reactions were terminated by adjustment to 50 mM EDTA and then mixing with an equal volume of 2 × Orange G loading buffer. The products were analyzed by electrophoresis at 4°C through a 20 cm 36% polyacrylamide gel containing 80 mM Tris-borate (pH 8.3) and 1 mM EDTA for 3 hours at 10 W constant power. The inositol polyphosphates were visualized by staining the gel with toluidine blue, as described above.

### Fission yeast strains

A list of strains used in this study is provided in Table S1.

### Spot tests of fission yeast growth

Cultures of *S. pombe* strains were grown in liquid ePMGT (enhanced pombe minimal glutamate with thiamine) (23) or YES (yeast extract with supplements) medium until *A*_600_ reached 0.3–0.8. The cultures were adjusted to an *A*_600_ of 0.1, and aliquots (3 µL) of serial fivefold dilutions were spotted to ePMGT or YES agar. The plates were photographed after incubation for 2 days at 34°C, 2 to 2.5 days at 30°C and 37°C, 4 days at 25°C, and 6 days at 20°C.

### Cell-surface acid phosphatase activity

Cells were grown at 30°C in YES medium. Aliquots of exponentially growing cultures were harvested, washed, and resuspended in water. To quantify acid phosphatase activity, reaction mixtures (200 µL) containing 100 mM sodium acetate (pH 4.2), 10 mM *p*-nitrophenylphosphate, and cells (ranging from 0.01 to 0.1 *A*_600_ units) were incubated for 5 minutes at 30°C. The reactions were quenched by addition of 1 mL of 1 M sodium carbonate, the cells were removed by centrifugation, and the absorbance of the supernatant at 410 nm was measured. Acid phosphatase activity is expressed as the ratio of *A*_410_ (*p*-nitrophenol production) to *A*_600_ (cells). The data are averages (±SEM) of at least three assays using cells from three independent cultures.

### Preparation of cell extracts and enrichment for inositol polyphosphates

Cultures of *S. pombe* strains were grown in liquid ePMGT medium at 30°C. Aliquots corresponding to 10 *A*_600_ units of exponentially growing cells (*A*_600_ between 0.6 and 0.8) were harvested by centrifugation. The cells were washed in ice-cold water and resuspended in 1 mL of 1 M perchloric acid. After snap-freezing in liquid nitrogen, the samples were stored at −80°C. The samples were thawed, mixed briefly by vortexing, and cell debris was removed by centrifugation at 16,200 g for 5 minutes at 4°C. Acid-extracted inositol polyphosphates in the supernatants were then purified using titanium dioxide beads (GL Sciences 5020–75000), as described by Wilson and Saiardi (42). In brief, the cell supernatants were mixed with TiO_2_ beads (4 mg per sample) that had been equilibrated in 1 M perchloric acid and incubated on a nutator for 20 minutes at 4°C. The TiO_2_ beads (plus bound material) were recovered by centrifugation (5,000 g for 1 min at 4°C) and washed twice with 1 M perchloric acid. Inositol polyphosphates were eluted from the beads in two cycles of resuspension in 200 µL of 2.8% ammonium hydroxide, rotation of the samples for 5 minutes at 4°C, and centrifugation (5,000 g for 1 minute). The combined eluates (400 µL per sample) were evaporated to dryness in a vacuum centrifuge (Savant SpeedVac) at 42°C for ~3 hours. The dried samples were stored at −80°C and resuspended in 30 µL of water immediately prior to analysis by CE-ESI-MS.

### Capillary electrophoresis electrospray ionization mass spectrometry (CE-ESI-MS)

The analyses were conducted as described (33, 34). Inositol polyphosphate levels were determined with an Agilent 7100 capillary electrophoresis (CE) system coupled to a triple–quadrupole tandem mass spectrometry Agilent 6495 c system. Ionization was performed with the help of an Agilent Jet Stream (AJS) electrospray ionization (ESI) source and a liquid coaxial interface from Agilent. Ionization spray was stabilized with sheath liquid containing a water–isopropanol (1:1) mixture and pumped with an Agilent 1200 isocratic LC pump and a 1:100 splitter to a flow rate of 10 µL/min. Cell extract samples were spiked with heavy (^13^C_6_) inositol polyphosphate standards (43, 44; provided by Dorothea Fiedler), and 20-nl aliquots of the samples were injected by applying a pressure of 100 mbar for 20 seconds. A bare fused silica capillary (length 100 cm; inner diameter 50 µm) was used for measurements. Ammonium acetate (35 mM, adjusted to pH 9.75 with ammonia solution) was used as the background electrolyte (BGE). By applying a voltage of +30 kV, a stable CE current of 22 µA was received. The gas temperature of the nebulizer was set to 150°C with a flow rate of 11 l/min and a pressure of 8 psi. Sheath gas flow was set at 8 L/min with a temperature of 175°C. Capillary voltage was set to 2,000 V and nozzle voltage to 2,000 V. 70 V was the negative high-pressure radio frequency, and the low-pressure radio frequency was 40 V. The multiple reaction monitoring (MRM) transitions (shown in Table S2) were optimized with the MassHunter Optimizer software.

## SUPPLEMENTAL MATERIAL

Supplemental Material mbio.01084-24-s0001.pdf Fig. S1; Tables S1 and S2.

ASM does not own the copyrights to Supplemental Material that may be linked to, or accessed through, an article. The authors have granted ASM a non-exclusive, world-wide license to publish the Supplemental Material files. Please contact the corresponding author directly for reuse.
