# Supplementary material for: Activities and genetic interactions of fission yeast Aps1, a Nudix-type inositol pyrophosphatase and inorganic polyphosphatase
Source: mBio. 2024 Jun 28;15(8):e01084-24. doi: 10.1128/mbio.01084-24 (PMC11323792; doi:10.1128/mbio.01084-24)
Supplement: Supplemental Material — Fig. S1; Tables S1 and S2. [file mbio.01084-24-s0001.pdf]

Supplemental Information

Activities and genetic interactions of fission yeast Aps1, a Nudix-type inositol pyrophosphatase and inorganic polyphosphatase

Shreya Ghosh, Ana M. Sanchez, Beate Schwer, Isabel Prucker, Nikolaus Jork, Henning J. Jessen, and Stewart Shuman

Figure S1

Tables S1 and S2

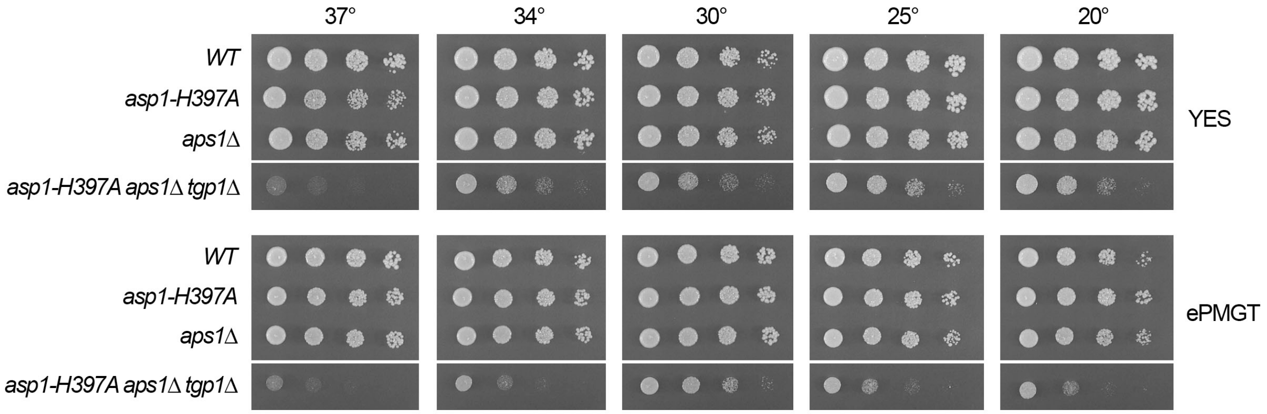

Figure S1. Growth defect of an *asp1-H397A aps1Δ tgp1Δ* triple-mutant. The indicated fission yeast strains were grown in liquid ePMGT medium at 30˚C. The cultures were adjusted to an A_{600} of 0.1 and aliquots (3 µl) of serial 5-fold dilutions were spotted to YES agar (top panel) or ePMGT agar (bottom panel) and incubated at the indicated temperatures. All four strains were spotted on the same YES or ePMGT plate. An extraneous strain was spotted between the *aps1Δ* and *asp1-H397A aps1Δ tgp1Δ* strains; the white space separating *aps1Δ* and *asp1-H397A aps1Δ tgp1Δ* signifies that the photographs were cropped to excise the intervening spottings.

Table S1. Fission yeast strains used in this study

Strain	Genotype	Source
JS77	*h- leu1-32 ura4-D18 his3-D1 ade6-m216*	1
JS78	*h+ leu1-32 ura4-D18 his3-D1 ade6-m210*	1
AS1884	*h- aps1Δ::kanMX*	2
AS1885	*h+ aps1Δ::kanMX*	2
AS2286	*h+ aps1Δ::hygMX*	2
AS2180	*h+ asp1-D333A::kanMX*	2
AS2181	*h+ asp1-H397A::kanMX*	2
AS2654	*h- asp1-H397A::natMX*	2
AS2183	*h+ asp1Δ::natMX*	2
BS272	*h- seb1-G476S::hygMX*	3
ASY89	*h- aps1-E89A-E93A::kanMX*	4
AS2303	*h+ siw14Δ::hygMX*	4
BS848	*h+ STF6::hygMX [asp1-W386*]*	5
BS858	*h+ STF9::hygMX [asp1-W493*]*	5
BS1038	*h+ kcs1-R332T::kanMX*	5
AGP237	*h- tgp1Δ::ura4+*	5
ASY152	*h- siw14Δ::hygMX aps1-E89A-E93A::kanMX*	This study
BS957	*h+ tgp1Δ::*$ura4^+$ *aps1Δ::hygMX*	This study
BS958	*h+ tgp1Δ::*$ura4^+$ *siw14Δ::hygMX*	This study
BS969	*h- siw14Δ::hygMX Δaps1::kanMX*	This study
BS971	*h+ tgp1Δ::*$ura4^+$ *siw14Δ::hygMX aps1Δ::kanMX*	This study
BS993	*h+ seb1-G476S::hygMX asp1-H397A::kanMX*	This study
BS995	*h- seb1-G476S::hygMX asp1-H397A::kanMX tgp1Δ::*$ura4^+$	This study
BS997	*h+ seb1-G476S::hygMX aps1Δ::kanMX*	This study
BS1003	*h- seb1-G476S::hygMX aps1Δ::kanMX tgp1Δ::*$ura4^+$	This study
BS1011	*h+ asp1-H397A aps1Δ::hygMX tgp1Δ::*$ura4^+$	This study

All strains are derived from the parental strains (JS77 and JS78). The strains are *leu1-32 ura4-D18 his3-D1* and either *ade6-m216 or ade6-m210*. The *asp1* alleles in the *STF* mutants are specified in brackets.

1. Pei Y, Du H, Singer J, St Amour C, Granitto S, Shuman S, Fisher RP. (2006) Cyclin-dependent kinase 9 (Cdk9) of fission yeast is activated by the CDK-activating kinase Csk1, overlaps functionally with the TFIIH-associated kinase Mcs6, and associates with the mRNA cap methyltransferase Pcm1 in vivo. *Mol Cell Biol* 26:777-788.
2. Sanchez AM, Garg A, Shuman S, Schwer B. (2019) Inositol pyrophosphates impact phosphate homeostasis via modulation of RNA 3' processing and transcription termination. *Nucleic Acids Res* 47: 8452-8469.
3. Schwer B, Garg A, Jacewicz A, Shuman S. (2021) Genetic screen for suppression of transcriptional interference identifies a gain-of-function mutation in Pol2 termination factor Seb1. *Proc Natl Acad Sci USA* 118: e2108105118.
4. Sanchez AM, Garg A, Schwer B, Shuman S. (2023) Duf89 abets lncRNA control of fission yeast phosphate homeostasis via its antagonism of precocious lncRNA transcription termination. *RNA* 29: 808-825.
5. Bednor L, Sanchez AM, Garg A, Shuman S, Schwer B. (2024) Genetic suppressor screen identifies Tgp1 (glycerophosphocholine transporter), Kcs1 (IP_6 kinase), and Plc1 (phospholipase C) as determinants of inositol pyrophosphate toxicosis in fission yeast. *mBio* 15: e03062-23.

Table S2. Parameters for multiple reaction monitoring transitions

	Precursor Ion	Product Ion	dwell	CE (V)	Cell Acc (V)	Polarity
$[^{13}C_6]$1,5-IP_8	411.9	362.8	80	9	1	Negative
1,5-IP_8	408.9	359.8	80	9	1	Negative
$[^{13}C_6]$1,5-IP_7	371.9	322.9	80	9	3	Negative
1,5-IP_7	368.9	319.9	80	9	3	Negative
$[^{13}C_6]IP_6$	331.9	486.9	80	13	4	Negative
IP_6	328.9	480.9	80	13	4	Negative
$[^{13}C_6]$2-OH IP_5	292.0	504.9	80	9	3	Negative
2-OH IP_5	289.0	498.9	80	9	3	Negative
